# SXT/R391 Integrative and Conjugative Elements (ICEs) Encode a Novel ‘*Trap-Door’* Strategy for Mobile Element Escape

**DOI:** 10.3389/fmicb.2016.00829

**Published:** 2016-05-31

**Authors:** Michael P. Ryan, Patricia Armshaw, J. Tony Pembroke

**Affiliations:** Molecular and Structural Biochemistry Group, Department of Chemical and Environmental Sciences, Materials and Surface Science Institute, University of LimerickLimerick, Ireland

**Keywords:** integrative conjugative elements (ICEs), UV sensitization, ‘*Trap-Door’* escape

## Abstract

Integrative conjugative elements (ICEs) are a class of bacterial mobile elements that have the ability to mediate their own integration, excision, and transfer from one host genome to another by a mechanism of site-specific recombination, self-circularisation, and conjugative transfer. Members of the SXT/R391 ICE family of enterobacterial mobile genetic elements display an unusual UV-inducible sensitization function which results in stress induced killing of bacterial cells harboring the ICE. This sensitization has been shown to be associated with a stress induced overexpression of a mobile element encoded conjugative transfer gene, *orf*43, a *tra*V homolog. This results in cell lysis and release of a circular form of the ICE. Induction of this novel system may allow transfer of an ICE, enhancing its survival potential under conditions not conducive to conjugative transfer.

## Introduction

Integrative conjugative elements (ICEs) are a class of diverse bacterial mobile elements that are characterized by their ability to mediate and encode all determinants for their own integration, excision, and transfer from one host genome to another by a mechanism of site-specific recombination, self-circularisation, and conjugative transfer (Taviani et al., 2009; Michael et al., 2012). In general the elements are mosaic in structure containing Phage-like integration systems, plasmid like transfer and maintenance systems and accumulated accessory genes with homology to a variety of organisms suggesting evolutionary accumulation via passage and residency in a variety of host organisms (Boltner et al., 2002; Pembroke et al., 2002). They are a major factor in the evolution of bacterial genomes allowing bacteria to rapidly acquire new phenotypic traits and adaptive functions such as resistance to antimicrobial compounds and heavy metals, virulence mechanisms, metabolic pathways (such as pathways for the degradation of xenobiotic pollutants) and the ability to resist bacteriophage infection (Ryan et al., 2009; Wozniak and Waldor, 2010; Guglielmini et al., 2011; Van Houdt et al., 2012). There are a growing number of ICEs being reported, many associated with multiple drug resistance and adaptive traits and archived in the ICE Berg Database^1^ (Bi et al., 2012).

The core set of genes required for a functionally active ICE are divided into three distinct modules known as the maintenance, conjugation, and regulation module (Wozniak and Waldor, 2009). The maintenance module contains the genes responsible for the stable integration of an ICE into a host genome and is defined by the presence of two genes *int* and *xis* (Storrs et al., 1991). The ICE integrase (*int*) is a recombinase which catalyzes the recombination reaction between specific recognition sites in a host genome (attB) and the circular ICE element usually called attP (located on the ICE; McGrath and Pembroke, 2004). In addition to the integrase most ICEs encode a recombination directionality factor (RDF) termed Xis which stimulates excision of ICE elements for transfer (Lewis and Hatfull, 2001; Abbani et al., 2005; O’Halloran et al., 2007; Wozniak and Waldor, 2010). ICEs transfer via the conjugation module and utilize conjugation mechanisms that are highly similar to those of conjugative plasmids. Gram negative ICEs typically use type IV secretion systems for conjugative transfer (Burrus and Waldor, 2004; Wozniak and Waldor, 2010; Guglielmini et al., 2011; Bellanger et al., 2014). ICE elements generally contain a regulatory module to control various aspects of ICE metabolism. ICE*St*1 and the SXT/R391 family have been found to have regulatory systems containing “phage-like” regulatory genes that are induced by DNA damaging agents (Beaber and Waldor, 2004; O’Halloran et al., 2007; Bellanger et al., 2008).

## The SXT/R391 Family of ICEs

The SXT/R391 family of ICEs is one of the largest of the ICE families with >100 elements being identified experimentally or bioinformatically from this group to date (Bi et al., 2012). This group was originally recognized following the sequencing of R391 (Boltner et al., 2002). Since then a large number of SXT/R391 elements have been identified based on similar mosaic structure while earlier elements dating back to the 1970s such as R997 and pMERPH have also been shown to be members of this group (McGrath et al., 2006). ICEs of the SXT/R391 family have been identified in both clinical (human and veterinary) and environmental isolates of Gammaproteobacteria in the main (Burrus and Waldor, 2004; Wozniak et al., 2009) which is significant as the integration site attB within the *prf*C gene (a 17 bp site at the 5′ end of the gene; Hochhut and Waldor, 1999; McGrath and Pembroke, 2004; Wozniak and Waldor, 2010) is highly conserved within this bacterial group (Armshaw and Pembroke, 2013c).

Since the start of the 1990s, SXT/R391 ICEs have been found to be widespread in both environmental and clinical *Vibrio cholerae* isolates from Asia and Africa (Burrus and Waldor, 2004; Spagnoletti et al., 2014). SXT/R391 ICEs have been found in all isolates recovered from cholera patients in Haiti (Ceccarelli et al., 2013) and are naturally occurring in many other enterobacteriaceae (Juiz-Rio et al., 2005; Pembroke and Piterina, 2006).

The ICE SXT (99 kb) is one of two archetypal members of the SXT/R391 family that was initially isolated from a multidrug resistant clinical isolate of *V. cholerae* O139 in India in 1992 (Beaber et al., 2002b). The second archetypal member of the SXT/R391 family is ICE R391 (89 kb), which was first discovered in 1967 in an isolate of *Providencia rettgeri* from South Africa (Boltner et al., 2002).

**FIGURE 1:**
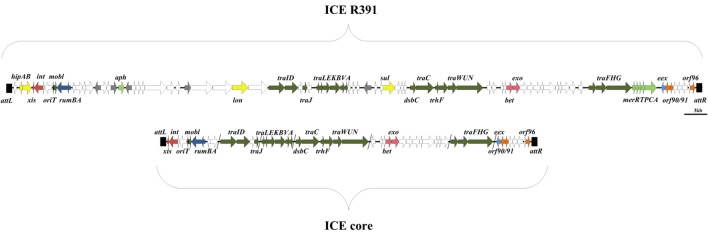
**Molecular map of the ICE R391 showing the location of the genes associated with the 89 kb element (Boltner et al., 2002; Armshaw and Pembroke, 2013c).** *orf*4 encoding the excisionase Jef/xis (O’Halloran et al., 2007) is located at the left end of the element in the integrated form. *Orf*43 here designated *tra*V is located in the center of the element within a cluster of ICE transfer genes (Armshaw and Pembroke, 2013a,b, 2015). Underneath the ICE core is illustrated containing genes that are common to all ICE elements (Wozniak et al., 2009).

All elements contain a conserved core set of genes (49 genes- 29 of known function and 20 hypothetical genes, see **Figure 1**) and sequences that facilitate regulation of element functions, their integration/excision and their conjugative transfer (Wozniak et al., 2009). Studies by Beaber et al. (2002a), Wozniak et al. (2009), and Lei et al. (2016) proposed that SXT/R391 family members contained five “hotspots” and five variable regions into which accessory (non-core) genes integrated at specific locations within the core genome. These genes code for proteins involved in antibiotic and heavy metal resistance, restriction modification systems, DNA repair systems, and many other functions.

## SOS Response Related to the SXT/R391 Family of ICEs

The SOS response is a global response to DNA damage in which the cell cycle is arrested and DNA repair and mutagenesis are induced. The system involves the RecA protein (Michel, 2005). In 2004 it was discovered that repression of *setC* and *setD* in ICE SXT (*orf*90 and 91 in ICE R391), which are homologous to transcriptional enhancers, resulted in induction of the SOS response, which promotes autoproteolysis of SetR (*Orf*96) (Beaber et al., 2004). *SetR*/*Orf*96 encodes a cI-like repressor protein with homology to the phage λ^434^ cI repressor. (Boltner et al., 2002) This in turn was found to upregulate the transfer of the ICE SXT element. Poulin-Laprade et al. (2015) demonstrated that expression of genes associated with the conjugative function of the SXT/R391 ICE family are tightly regulated by *SetR/Orf96*. This represses the expression of the transcriptional enhancer genes *setC/orf90* and *setD/orf91*. The products of these genes activate transcription of ICE encoded *int*, *jef* (*xis*) and conjugation-associated operons (O’Halloran et al., 2007; Poulin-Laprade et al., 2015).

## Investigating UV Sensitization of the SXT/R391 Family

Members of the ICE SXT/R391 family have been shown to induce an unusual UV-inducible sensitization function following DNA damage (Pembroke and Stevens, 1984; McGrath et al., 2006). In the early 1980s it was noted that the presence of R391 and R997 sensitized a strain of *Escherichia coli* AB1157 to UV irradiation, significantly decreasing post-irradiation cell survival rates. It was thought that this sensitization effect was most likely due to interference with one of the RecA^∗^-induced DNA damage repair pathways of *E. coli* as the effect was shown to be *recA*-dependent but *lexA*-independent (Pembroke and Stevens, 1984). The mechanism could not be elucidated at the time and no rationale as to why the R391 element (ICE R391) should possess this effect on host cells was proposed.

Analysis of the nature of UV sensitization associated with ICE R391 was then investigated again using a structured deletion library of the entire ICE (Armshaw and Pembroke, 2013a,b). It appeared that deletion of ICE encoded *orf*90 and 91 (the R391 transcriptional enhancers) and a conjugative transfer gene *orf*43 (encoding a *Tra*V homolog) abolished the induced sensitization. This was interesting as *orf*43 was found to be one of the most conserved of the “core genes” of the SXT/R391 family with all homologs (found to date) being ≥98% similar, indicating that it may be under evolutionary pressure to stay conserved. *Orf*43 is a TraV homolog which forms part of the mating pore during conjugation (Alvarez-Martinez and Christie, 2009). Characterization of how this gene is expressed revealed that UV and other DNA damaging agents induced the hosts *rec*A gene who’s product *Rec*A^∗^ cleaved the ICE R391 repressor *orf*96. Cleavage of *orf*96 resulted in induction of *orf*90 and 91 (Armshaw and Pembroke, 2013c; Poulin-Laprade et al., 2015).

It had previously been demonstrated that induction of *orf*90/91 resulted in the upregulation of expression of *orf*4 which encodes for the ICE R391 excisionase, Jef (J excisionase). Induction of Jef, which is also known as Xis (O’Halloran et al., 2007), resulted in an increased copy number of the circular transfer intermediate of ICE R391 and hence an increased conjugation rate to recipients cells (O’Halloran et al., 2007). This was also shown by Poulin-Laprade et al. (2015).

It was of interest to then examine if this expression hierarchy, which resulted in induction of *orf*43 (Armshaw and Pembroke, 2013a, 2015), was associated with Jef/Xis and indeed what was the mechanism of sensitization resulting from *orf*43 induction.

Cloning and controlled expression of *orf*43 was utilized initially to probe the nature of the ‘UV sensitization’ (Armshaw and Pembroke, 2015). Upon overexpression, as occurs upon UV induction controlled by *orf90*/*91*, increased cell permeability and cell lysis was observed (Armshaw and Pembroke, 2015) consistent with increased pore formation within the ICE R391 cell. Transmission electronic microscopy (TEM) analysis revealed significant cell lysis (Armshaw and Pembroke, 2015) consistent with cell death and the sensitization observed following native *orf*43 induction.

## ‘*Trap-Door*’ Escape

Although, the significance of such a detrimental effect was not immediately obvious when the two phenomenon (increased circular form of the mobile element and cell bursting) were linked, a testable hypothesis emerged. On the one hand DNA damage induces excision of the element (O’Halloran et al., 2007; Poulin-Laprade et al., 2015) while at the same time causing cell lysis. It was reasoned that this lysis might allow for a ‘*trap-door’* effect whereby the circular intermediate (the excised ICE element), might escape in the absence of a functional conjugative mechanism such as might be the case in severely damaged cells. This hypothesis was tested using specific deletion mutants of ICE R391 that were deficient in conjugative transfer (Armshaw and Pembroke, 2013a). These strains were unable to undergo conjugative transfer at any detectable levels. However, upon *orf*43 induction as occurs in cells with DNA damage that contained conjugative defective ICE R391, it was determined that low levels of detectable transfer could be restored (Armshaw and Pembroke, 2015). Effectively, the apparent UV sensitization/DNA damage allows the formation of a ‘trap-door’ to allow ICE survival upon significant DNA damage to the host cell. This mechanism could allow SXT/R391 ICEs, which are missing necessary conjugative genes (possible examples of these were identified by Spagnoletti et al., 2014, however, alternative explanations for selective loss of conjugation functions post-transfer may apply) to transfer via the ‘trap-door’ mechanism at low levels. The elements are then taken up by other cells in “apparent transformation” In Armshaw and Pembroke (2015); experiments were carried out with gene knockouts. The conjugation apparatus genes *orf*40–*orf*44 were knocked out. Transfer rates of <10^-10^ were found. When an expression plasmid with *orf43* was added to this knockout transfer was restored at rates of 1.54 × 10^-7^ (without UV) and 1.01 × 10^-6^ (with UV). Addition of DNase abolished transfer (Transfer rates of <10^-10^). This indicated that some form of transformation must have occurred.

**FIGURE 2:**
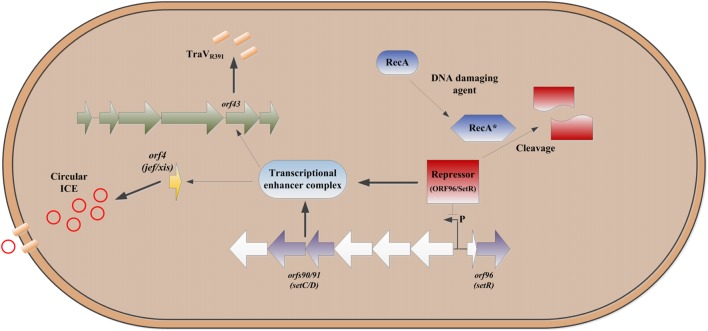
**Outline of the escape mechanism proposed for ICE’s of the SXT/R391 group.** DNA damage causes cleavage of *orf*96 leading to upregulation of expression of *orf*90/91 (Beaber et al., 2004), which in turns leads to induction of *orf*4 and production of the ICE excisionase (Jef/Xis) which results in increased levels of the circular form of the ICE (O’Halloran et al., 2007). UV irradiation also results in increased expression of *orf*43 which results in overproduction of the ICE *Tra*V homolog (Armshaw and Pembroke, 2013a,b, 2015) which results in increased cell porosity and cell bursting providing an escape or ‘trap-door’ for the ICE.

This mechanism (**Figure 2**) could also provide a back-up escape route to allow conjugation levels discussed above, so that if the cell were so damaged that conjugation cannot take place then a secondary escape mechanism would be available to the element. This appears to be a novel mechanism, which we term a ‘trap-door,’ of mobile element survival and presumably occurs in tandem with formation of circular transfer intermediate, which is subsequently released as a result of pore formation and cell lysis making the element available for transformation allowing for some ICE survival in extreme environments. This proposed mechanism is so far unique to the SXT/R391 family and no comparable escape mechanism has been found in other mobile genetic elements thus far.

## Summary of Mechanism

1. DNA of the bacterial cell is damaged (by UV, chemical DNA damaging agent such as mitomycin C, etc.) creating single stranded DNA which stimulates RecA to become its active form RecA^∗^ (O’Reilly and Kreuzer, 2004).
2. RecA^∗^ cleaves the putative ICE SXT/R391 *setCD*/*orf90-91* repressor protein *setR/orf96* (Pembroke and Stevens, 1984; Beaber et al., 2004; McGrath et al., 2005).
3. Cleavage of the putative repressor protein *setR/orf96* causes up-regulated expression of the putative transcriptional activator complex encoded by *setCD*/*orf90-91* (Beaber et al., 2004).
4. The putative transcriptional activator complex encoded by *setCD*/*orf90-91* binds a regulatory region upstream of *orf*43, upregulating the expression of TraV_R391_ (*orf*43) in a manner that is cytotoxic to the host cell (Armshaw and Pembroke, 2013b, 2015; Poulin-Laprade et al., 2015).
5. The putative transcriptional activator complex encoded by *setCD*/*orf90-91* also binds a regulatory region upstream of *orf*4 which encodes the Jef protein, a protein that mediates the excision of the integrated SXT/R391 ICE from the bacterial chromosome (O’Halloran et al., 2007; Poulin-Laprade et al., 2015).
6. The cytotoxic effect is the result of damage to the cells outer membrane caused via the action of TraV_R391_ which causes the formation of pores in the membrane which allow escape of the excised SXT/R391 ICE element (the ‘*trap–door’*) and the death of the cell (Armshaw and Pembroke, 2013b, 2015) (**Figure 2**).
7. The SXT/R391 ICE element is then available to be taken (via ‘apparent transformation’) into other bacterial cells (Armshaw and Pembroke, 2015)

## Author Contributions

All authors listed, have made substantial, direct and intellectual contribution to the work, and approved it for publication.

## Conflict of Interest Statement

The authors declare that the research was conducted in the absence of any commercial or financial relationships that could be construed as a potential conflict of interest.

## Footnotes

1 http://db-mml.sjtu.edu.cn/ICEberg/
